# Hospital-Based Proportion of Diabetic Retinopathy and Associated Risk Factors Among Patients With Type 2 Diabetes Mellitus

**DOI:** 10.7759/cureus.107098

**Published:** 2026-04-15

**Authors:** Nayef Alswaina, Fatimah M Alayed, Meshari A Alharbi, Jenan K Alqurishi, Rola Alradaddi, Razan K Alduraibi, Abdulrahman S Alqazlan

**Affiliations:** 1 Department of Ophthalmology, Qassim University, Buraydah, SAU; 2 College of Medicine, Qassim University, Qassim, SAU; 3 College of Medicine, Qassim University, Buraydah, SAU

**Keywords:** diabetic retinopathy, retinopathy, risk factors, saudi arabia, severity

## Abstract

Introduction

Diabetic retinopathy (DR) is a leading cause of blindness in individuals with diabetes, and its prevalence is increasing worldwide. This study aimed to explore the relationship between various demographic, clinical, and biochemical factors and the proportion of DR in patients with diabetes at Qassim University Hospital, Qassim, Saudi Arabia.

Methods

A retrospective cross-sectional study was conducted at Qassim University Hospital, Saudi Arabia, including 370 adult patients with type 2 diabetes mellitus (T2DM). Data were extracted from electronic medical records, including age, gender, duration of diabetes, HbA1c, BMI, comorbidities, medication type, and blood pressure status. Diabetic retinopathy and macular edema were diagnosed based on ophthalmologic evaluation. Statistical analyses included descriptive statistics, Chi-squared tests, independent t-tests, and one-way ANOVA, with significance set at p<0.05.

Results

A total of 370 patients were included in the study. The mean age of the participants was 55.77 ± 13.98 years, and 54.1% (n=200) were male. The proportion of any diabetic retinopathy was 28.6% (n=106), including 11.6% (n=43) mild non-proliferative diabetic retinopathy (NPDR), 7.8% (n=29) moderate NPDR, 1.9% (n=7) severe NPDR, and 7.3% (n=27) PDR. Macular edema was present in 13.0% (n=48). Patients with DR were significantly older (p<0.001) and had longer diabetes duration (p<0.001) and higher HbA1c (p<0.001) compared with those without DR. Insulin therapy (p<0.001) and hypertension (p=0.024) were also significantly associated with DR. No significant associations were found between DR and comorbidities such as dyslipidemia, hypothyroidism, asthma, BPH, ischemic heart disease, obstructive sleep apnea, chronic kidney disease, or gastrointestinal disorders. ANOVA showed significant differences across DR severity groups for age (p=0.001), duration of diabetes (p<0.001), and HbA1c (p<0.001). Multivariate analysis identified age, duration of diabetes, and HbA1c as independent predictors of diabetic retinopathy (p<0.05).

Conclusion

Diabetic retinopathy affects nearly one-third of patients with T2DM in this population and is strongly associated with older age, longer disease duration, poor glycemic control, insulin use, and hypertension. These findings highlight the importance of early screening and risk factor management. However, the retrospective design and single-center setting may limit generalizability.

## Introduction

Diabetes mellitus (DM) is becoming increasingly prevalent [1]. The International Diabetes Federation estimated that 463 million adults were living with diabetes in 2019, with projections indicating this figure will rise to 700 million by 2045 [2]. The Middle East and North Africa region has experienced a particularly sharp increase in diabetes prevalence, driven by rapid urbanization, sedentary lifestyles, and changing dietary habits [3]. Within this region, the Kingdom of Saudi Arabia bears a substantial diabetes burden, ranking second in the Middle East and seventh globally for diabetes prevalence. Current estimates indicate that approximately seven million Saudi adults are diagnosed with diabetes, with an additional three million in the prediabetes stage [4]. Recent national data confirm that diabetes prevalence remains high, with a cross-sectional analysis reporting a prevalence of 27% among Saudi adults [5], while population-based projections estimate that the number of individuals living with diabetes will increase from 2.69 million in 2020 to 4.21 million by 2030 [6].

Diabetic retinopathy (DR) is a serious complication of microvascular changes that occur in most body systems in patients with diabetes over time. Diabetic retinopathy is the most common factor contributing to new-onset blindness in patients aged 20-74 years. During the first 20 years of the disease, nearly all patients with type 1 DM and up to 60% of patients with type 2 DM have developed retinopathy at some point [7]. Within Saudi Arabia, previous studies have reported considerable variation in DR prevalence across different regions. A population-based study in Taif reported that 36% of diabetic patients developed some form of DR [8], while a study in Al-Madinah found a similar estimate of 36.1% [9], and another in Al-Hasa reported a prevalence of 30% [10]. A recent systematic review and meta-analysis encompassing 11 studies published between 2006 and 2019 estimated the overall pooled prevalence of DR in Saudi Arabia at 31% (95% CI: 24-39%), with substantial heterogeneity across studies (I²=99%) and individual study estimates ranging from 16.7% to 69.8% [11]. This wide variation is attributed to differences in study design, population characteristics, duration of diabetes, and glycemic control. Despite these national estimates, data specific to the Al-Qassim region remain limited. Qassim University Hospital serves a diverse population from both urban and rural areas within the region, yet the current prevalence of DR and its associated risk factors among diabetic patients in this catchment area have not been well characterized. Regional assessments are particularly important given that healthcare access, screening practices, and patient awareness may differ substantially across Saudi Arabia's provinces. A recent study from the same institution focused on newly diagnosed diabetic patients reported a low DR prevalence of 7.8%, reflecting the short diabetes duration in that cohort [12]. However, no recent study has comprehensively evaluated DR prevalence across the broader spectrum of patients with established type 2 diabetes in this region. Understanding the regional prevalence and risk factors for DR is essential to guide targeted public health initiatives and resource allocation. The present study aimed to determine the prevalence of diabetic retinopathy and identify its associated risk factors among patients with type 2 diabetes mellitus (T2DM) attending Qassim University Hospital.

## Materials and methods

Study design and setting

This retrospective cross-sectional study was conducted at Qassim University Hospital, a tertiary care and teaching institution located in the Qassim region of Saudi Arabia. The hospital serves a diverse diabetic population from both urban and rural areas. The study was carried out over a seven-month period from January to July 2024.

Study population

The study population comprised patients diagnosed with type 2 diabetes mellitus (T2DM) who were regularly attending the diabetes outpatient clinics at Qassim University Hospital. A total of 370 patients were included in the final analysis. Eligibility was limited to adult patients aged 18 years and above who had a confirmed diagnosis of T2DM and complete medical records containing all relevant variables, including demographic details, duration of diabetes, HbA1c levels, comorbidities, and ophthalmologic findings. Patients with type 1 DM, incomplete medical records, or ocular diseases unrelated to diabetic retinopathy were excluded from the study. A non-probability consecutive sampling technique was used to include all eligible patients meeting the inclusion criteria during the study period.

Sample size determination

The sample size was determined using Cochran's formula based on an estimated diabetic population of approximately 10,000 patients attending the diabetes clinics at Qassim University Hospital. By applying a 95% confidence interval and a 5% margin of error, the minimum required sample size was calculated to be 370 participants. The final sample met this requirement, ensuring adequate statistical power for the analyses.

Data collection procedure

Data were retrospectively collected from patients' electronic medical records after obtaining institutional approval. The information retrieved included demographic characteristics such as age, gender, occupation, and family history of diabetes, as well as clinical variables including duration of diabetes, age at onset, body mass index (BMI), blood pressure status, and HbA1c levels. Treatment details were also reviewed, including the type of anti-diabetic medication used, categorized as either oral hypoglycemic agents or insulin therapy, with or without additional drugs. Comorbidities such as hypertension, dyslipidemia, hypothyroidism, ischemic heart disease, asthma, benign prostatic hyperplasia, chronic kidney disease, and other chronic illnesses were also recorded. Ophthalmologic findings were extracted from documented eye examinations, specifically focusing on the presence and grade of diabetic retinopathy and the presence or absence of macular edema. The grading of retinopathy was determined according to the Early Treatment Diabetic Retinopathy Study (ETDRS) classification, which categorizes diabetic retinopathy into mild, moderate, and severe non-proliferative diabetic retinopathy (NPDR) and proliferative diabetic retinopathy (PDR) [13].

Statistical analysis

All data were entered and analyzed using the Statistical Package for the Social Sciences (SPSS) version 26.0 (IBM Inc., Armonk, NY, USA). Descriptive statistics were computed for all study variables. Continuous variables such as age, duration of diabetes, and HbA1c levels were expressed as means and standard deviations, while categorical variables such as gender, presence of retinopathy, and comorbidities were summarized as frequencies and percentages. To determine associations between categorical variables, the Chi-squared test or Fisher's exact test was used where appropriate. Independent t-tests and one-way analysis of variance (ANOVA) were used to compare mean differences between groups where appropriate. Binary logistic regression analysis was performed to identify independent predictors of diabetic retinopathy after adjusting for potential confounders. Variables that were clinically relevant or showed statistical significance in univariate analysis were included in the model. Adjusted odds ratios (ORs) with 95% confidence intervals (CIs) were calculated. The normality of continuous variables was assessed using the Shapiro-Wilk test and visual inspection of histograms and Q-Q plots. A p-value of less than 0.05 was considered statistically significant.

Ethical considerations

Ethical approval for this study was obtained from the Regional Research Ethics Committee, which is registered at the National Committee of Bio & Med. Ethics (NCBE) (Approval number: 607/45/1421). The study was conducted in accordance with the principles outlined in the Declaration of Helsinki. Due to the retrospective nature of the study, the requirement for informed consent was waived by the ethics committee. All patient data were anonymized prior to analysis to ensure confidentiality and compliance with data protection standards.

## Results

A total of 370 patients were included in this study. Of these, 54.1% (n=200) were male, and 45.9% (n=170) were female. The mean age of participants was 55.77 ± 13.98 years, while the mean duration of diabetes was 11.24 ± 9.26 years. The average HbA1c level was 7.76 ± 1.75%, and the mean BMI was 29.81 ± 5.92 kg/m². Most patients, 70.3% (n=260), were using oral hypoglycemic agents, whereas 29.7% (n=110) were receiving insulin with or without oral agents. Hypertension was present in 44.6% (n=165) of the sample, and 56.8% (n=210) had at least one comorbidity. Detailed demographic and clinical characteristics are presented in Table 1.

**Table 1:** Demographic and clinical characteristics of the study population (N=370)

Variable	Category / mean ± SD	n	%
Gender	Male	200	54.1
	Female	170	45.9
Age (years)	55.77 ± 13.98	—	—
Duration of diabetes (years)	11.24 ± 9.26	—	—
HbA1c (%)	7.76 ± 1.75	—	—
BMI (kg/m²)	29.81 ± 5.92	—	—
Total cholesterol (mmol/L)	4.48 ± 1.76	—	—
Anti-diabetic medication	Oral hypoglycemic agents	260	70.3
	Insulin ± oral agents	110	29.7
Blood pressure status	Hypertensive	165	44.6
	Normotensive	205	55.4
Nephropathy	Present	28	7.6
Comorbidities (any)	Present	210	56.8
Most common comorbidities	Dyslipidemia	148	40
	Hypothyroidism	27	7.3
	Asthma	14	3.8
	Benign prostate hyperplasia	10	2.7
	Ischemic heart disease	7	1.9
	Obstructive sleep apnea	2	0.5
	Chronic kidney disease	3	0.8
	Bone disorders	4	1.1
	Gastrointestinal disorders	3	0.8
	Other comorbidities	20	5.4

**Figure 1:**
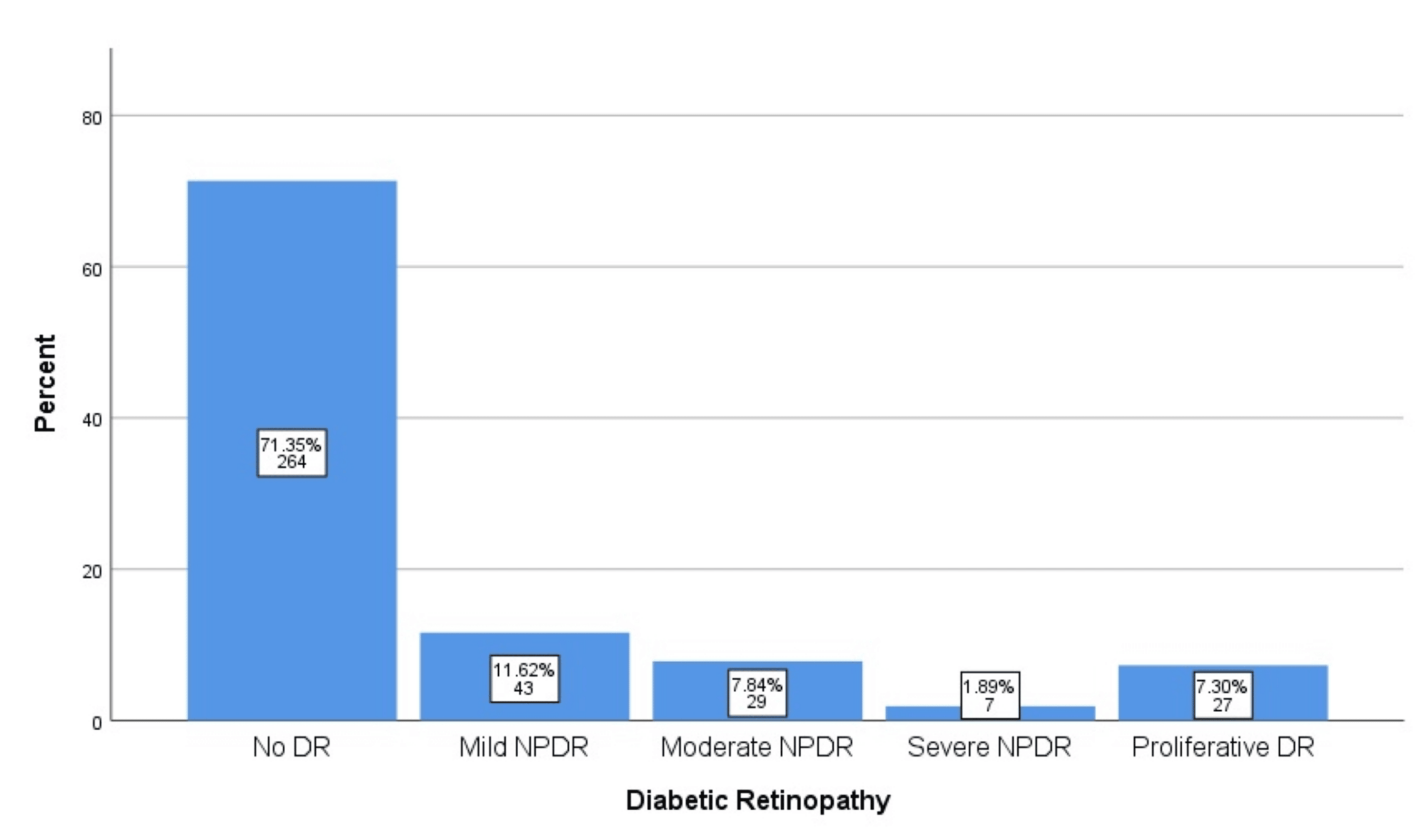
Proportion of diabetic retinopathy and macular edema DR - diabetic retinopathy; NPDR - non-proliferative diabetic retinopathy

Diabetic retinopathy (DR) was identified in 28.6% (n=106) of the patients. Among the affected individuals, 11.6% (n=43) had mild NPDR, 7.8% (n=29) had moderate NPDR, 1.9% (n=7) had severe NPDR, and 7.3% (n=27) had proliferative DR. Macular edema was present in 13.0% (n=48) of the sample. A complete distribution of retinopathy grades is provided in Figure 1.

Comparative analysis showed no significant association between gender and the presence of DR (p=0.945). However, patients with DR were significantly older (60.61 ± 11.76 years) than those without DR (53.83 ± 14.34 years, p<0.001). The duration of diabetes was also significantly longer among patients with DR (17.05 ± 9.60 years) compared with those without (8.90 ± 8.02 years, p<0.001). Glycemic control differed significantly between groups, with higher HbA1c levels observed in patients with DR (8.50 ± 1.87% vs. 7.46 ± 1.61%, p<0.001). BMI did not differ significantly (p=0.278). DR was more common among patients on insulin therapy (46.4%) than among those on oral agents alone (21.2%, p<0.001). Hypertension was also significantly associated with DR, with 34.5% (n=57) of hypertensive patients affected compared to 23.9% (n=49) of normotensive patients (p=0.024). These findings are summarized in Table 2.

**Table 2:** Association between diabetic retinopathy and demographic/clinical variables *Statistical significance at p<0.05 DR - diabetic retinopathy

Variable	Category / mean ± SD	No DR n (%)	With DR n (%)	Statistic	p-value
Gender	Male	143 (71.5)	57 (28.5)	χ² = 0.005	0.945
	Female	121 (71.2)	49 (28.8)		
Age (years)	Mean ± SD	53.83 ± 14.34	60.61 ± 11.76	t = -4.57	<0.001*
Duration of diabetes (years)	Mean ± SD	8.90 ± 8.02	17.05 ± 9.60	t = -8.27	<0.001*
HbA1c (%)	Mean ± SD	7.46 ± 1.61	8.50 ± 1.87	t = -4.76	<0.001*
BMI (kg/m²)	Mean ± SD	30.04 ± 6.10	29.31 ± 5.11	t = 1.09	0.278
Medication	Oral agents	205 (78.8)	55 (21.2)	χ² = 24.03	<0.001*
	Insulin ± oral agents	59 (53.6)	51 (46.4)		
Blood pressure	Hypertensive	108 (65.5)	57 (34.5)	χ² = 5.07	0.024*
	Normotensive	156 (76.1)	49 (23.9)		

No significant associations were found between DR and any of the comorbidities assessed, including dyslipidemia (p=0.280), hypothyroidism (p=0.443), asthma (p=0.231), benign prostatic hyperplasia (p=0.186), ischemic heart disease (p=0.996), obstructive sleep apnea (p=0.369), chronic kidney disease (p=0.857), bone disorders (p=0.342), or gastrointestinal disorders (p=0.857). Full comparisons are presented in Table 3.

**Table 3:** Association between diabetic retinopathy and comorbidities Statistical significance at p<0.05 DR - diabetic retinopathy

Comorbidity	No DR n (%)	With DR n (%)	Statistic	p-value
Dyslipidemia	101 (38.3)	47 (44.3)	χ² = 1.17	0.28
Hypothyroidism	21 (8.0)	6 (5.7)	χ² = 0.59	0.443
Asthma	8 (3.0)	6 (5.7)	χ² = 1.44	0.231
Benign prostatic hyperplasia	9 (3.4)	1 (0.9)	χ² = 1.75	0.186
Ischemic heart disease	5 (1.9)	2 (1.9)	χ² = 0.00	0.996
Obstructive sleep apnea	2 (0.8)	0 (0.0)	χ² = 0.81	0.369
Chronic kidney disease	2 (0.8)	1 (0.9)	χ² = 0.03	0.857
Bone disorders	2 (0.8)	2 (1.9)	χ² = 0.90	0.342
Gastrointestinal disorders	2 (0.8)	1 (0.9)	χ² = 0.03	0.857
Other comorbidities	16 (6.1)	4 (3.8)	χ² = 0.77	0.379

Analysis of variance across retinopathy severity levels demonstrated significant differences in age (p=0.001), duration of diabetes (p<0.001), and HbA1c levels (p<0.001). BMI did not differ significantly across severity groups (p=0.492). These results are shown in Table 4.

**Table 4:** Comparison of clinical characteristics across retinopathy severity levels (one-way ANOVA) *Statistical significance at p<0.05 DR - diabetic retinopathy; NPDR - non-proliferative diabetic retinopathy; PDR - proliferative diabetic retinopathy

Variable	No DR (n=264) mean ± SD	Mild NPDR (n=43) mean ± SD	Moderate NPDR (n=29) mean ± SD	Severe NPDR (n=7) mean ± SD	PDR (n=27) mean ± SD	F-value	p-value
Age (years)	53.83 ± 14.34	61.79 ± 11.01	60.72 ± 10.89	59.00 ± 10.94	59.04 ± 14.20	4.44	0.001*
Duration of Diabetes (years)	8.90 ± 8.02	15.14 ± 9.59	18.69 ± 9.53	13.43 ± 8.40	19.26 ± 9.61	35.32	<0.001*
HbA1c (%)	7.46 ± 1.61	8.22 ± 1.53	8.18 ± 1.54	9.47 ± 2.82	9.05 ± 2.25	6.17	<0.001*
BMI (kg/m²)	30.00 ± 6.22	30.28 ± 4.87	29.04 ± 4.84	27.29 ± 5.30	28.60 ± 5.62	0.86	0.492

Binary logistic regression analysis was performed to identify independent predictors of diabetic retinopathy. The overall model was statistically significant (χ²=88.81, p<0.001) and explained approximately 30.6% of the variance (Nagelkerke R²=0.306). The model demonstrated good fit as indicated by the Hosmer-Lemeshow test (p=0.610). Increasing age (p=0.012), longer duration of diabetes (p<0.001), and higher HbA1c levels (p<0.001) were found to be independent predictors of diabetic retinopathy. In contrast, gender (p=0.137), BMI (p=0.383), anti-diabetic medication use (p=0.075), and hypertension (p=0.552) were not significantly associated after adjustment (Table 5).

**Table 5:** Multivariate logistic regression analysis of factors associated with diabetic retinopathy

Variable	B	SE	Adjusted OR (Exp B)	95% CI	p-value
Age	0.028	0.011	1.03	1.01-1.05	0.012
Duration of diabetes mellitus	0.075	0.017	1.08	1.05-1.12	<0.001
HbA1c	0.3	0.075	1.35	1.17–1.56	<0.001
Gender (Male)	0.424	0.285	1.53	0.87-2.70	0.137
BMI	-0.021	0.024	0.98	0.93-1.03	0.383
Anti-diabetic medication	-0.567	0.319	0.57	0.30-1.08	0.075
Hypertension	0.167	0.281	1.18	0.68-2.05	0.552

## Discussion

The present study demonstrated that diabetic retinopathy (DR) affects 28.6% of patients with type 2 diabetes mellitus in our study population. This finding is consistent with regional and global estimates, supporting the substantial burden of DR in diabetic populations. A recent meta-analysis conducted in Saudi Arabia reported an overall pooled prevalence of approximately 31%, with considerable variability across regions and study designs [11]. Similarly, global estimates suggest that DR affects approximately 30-40% of individuals with diabetes, highlighting its significance as a major public health concern [14]. The similarity of our findings to these estimates strengthens the external validity of the study while reflecting the ongoing burden of DR in Saudi Arabia.

Another study estimated the global prevalence of DR to be 27.0% [15], leading to 0.4 million people with blindness [16]. In addition, a pooled analysis reported the prevalence of diabetic retinopathy in Africa to be 30.2-31.6% [17]. A recent study in Egypt reported that the prevalence of diabetic retinopathy was 46.4% in patients with diabetes [18]. In Saudi Arabia, a previous study reported a prevalence of 19.7% using data from the Saudi National diabetic registry [19]. In addition, a systematic review of 12 studies conducted in Saudi Arabia reported a prevalence of 6.25-88.1% [20].

Consistent with existing literature, increasing age, longer duration of diabetes, and poor glycemic control were significantly associated with DR in this study [21]. These factors have been widely recognized as key determinants of microvascular complications, including DR, due to their cumulative effect on retinal microvasculature. Chronic hyperglycemia leads to oxidative stress, inflammation, and endothelial dysfunction, ultimately resulting in capillary damage and retinal ischemia [22]. Previous studies conducted in Saudi Arabia have similarly identified these factors as major contributors to DR development [11].

The observed association between insulin therapy and DR in this study warrants careful interpretation. Rather than indicating a direct causal relationship, insulin use likely reflects underlying disease severity. Patients requiring insulin are often those with longer disease duration, poorer glycemic control, or advanced disease states, all of which are independently associated with DR. This phenomenon has been reported in previous studies, where insulin therapy acts as a surrogate marker for more advanced or poorly controlled diabetes rather than a direct risk factor [23].

Hypertension showed a significant association in univariate analysis; however, this association was not retained after adjustment in multivariate analysis. Elevated blood pressure contributes to increased shear stress on retinal vessels, leading to endothelial dysfunction, breakdown of the blood-retinal barrier, and enhanced vascular permeability [24]. These changes accelerate microvascular damage and promote the progression of DR. Several studies have highlighted hypertension as an important modifiable risk factor, emphasizing the role of blood pressure control in preventing or delaying DR progression [25].

In contrast, dyslipidemia was not significantly associated with DR in this study. This finding is in line with some previous reports but remains controversial, as the relationship between lipid abnormalities and DR is complex and not fully understood. Variability in lipid control, differences in study populations, and the influence of lipid-lowering therapies may partly explain these inconsistent findings. Additionally, lipid abnormalities may play a more prominent role in specific manifestations such as diabetic macular edema rather than DR overall, which could contribute to the lack of significant association observed.

Compared with other regional studies, the proportion and associated risk factors identified in this study align with findings from Saudi Arabia and neighboring regions, where the DR proportion in T2DM varies widely depending on study design, population characteristics, and screening practices [26]. Differences in healthcare access, early detection programs, and patient awareness may contribute to these variations. Internationally, similar patterns have been observed, with higher prevalence reported in populations with longer disease duration and suboptimal glycemic control [27]. The findings from multivariate analysis suggest that the observed associations for some variables in univariate analysis may be confounded by other factors, highlighting the importance of adjusted analyses.

The findings of this study have important implications for clinical practice. First, they highlight the need for regular screening for retinopathy and other complications in patients with diabetes, particularly those with poor glycemic control and long-standing diabetes. Early detection and intervention are critical to prevent the progression of retinopathy and preserve vision in patients with diabetes. Second, this study underscores the importance of comprehensive management of diabetes, including stringent glycemic control, blood pressure management, and regular monitoring for complications. Given the high prevalence of comorbidities, such as hypertension and dyslipidemia, in diabetic patients, a multidisciplinary approach to care is essential to address various risk factors and prevent complications. Finally, further research is needed to better understand the complex relationships among retinopathy, comorbidities, and other diabetes-related complications. Prospective studies with larger sample sizes and longer follow-up periods could provide more insight into the risk factors for the progression of retinopathy and the effectiveness of different interventions in preventing complications.

This study has several limitations that should be acknowledged. First, the retrospective cross-sectional design limits the ability to establish temporal relationships or infer causality between the observed variables and diabetic retinopathy. Second, the study was conducted at a single tertiary care center, which may limit the generalizability of the findings to other populations or healthcare settings. Third, the use of electronic medical records as the primary data source introduces the possibility of information bias, as data completeness and accuracy depend on routine clinical documentation. Additionally, ophthalmologic assessments were performed by different clinicians as part of routine care, and interobserver variability could not be assessed, which may have introduced measurement bias. Although multivariate binary logistic regression analysis was performed to adjust for measured confounders, the possibility of residual confounding cannot be excluded. Important variables such as medication adherence, socioeconomic status, lifestyle factors, and dietary habits were not available in the medical records and therefore could not be included in the analysis. Furthermore, only patients with complete records were included, which may have introduced selection bias. These limitations should be considered when interpreting the findings, and future prospective, multicenter studies with standardized data collection are recommended to validate and expand upon these results.

## Conclusions

This study demonstrated that DR is a common microvascular complication among patients with type 2 diabetes mellitus in this population, affecting nearly one-third of participants. Increasing age, longer duration of diabetes, poor glycemic control, insulin therapy, and hypertension were significantly associated with the presence of retinopathy. These findings highlight the importance of targeted retinal screening, particularly for high-risk patients, and reinforce the need for optimized glycemic and blood pressure control. From a public health perspective, strengthening screening programs and patient education initiatives may help reduce the burden of vision-threatening complications. However, given the single-center nature of the study, these findings should be interpreted within the context of the study population. Future prospective multicenter studies are recommended to validate these findings and better establish causal relationships.
